# Red cell distribution width/albumin ratio and 90-day mortality after burn surgery

**DOI:** 10.1093/burnst/tkab050

**Published:** 2022-01-27

**Authors:** Young Joo Seo, Jihion Yu, Jun-Young Park, Narea Lee, Jiwoong Lee, Ji Hyun Park, Hee Yeong Kim, Yu-Gyeong Kong, Young-Kug Kim

**Affiliations:** Department of Anesthesiology and Pain Medicine, Hangang Sacred Heart Hospital, Hallym University College of Medicine, Seoul 07247, Republic of Korea; Department of Anesthesiology and Pain Medicine, Asan Medical Center, University of Ulsan College of Medicine, Seoul 05505, Republic of Korea; Department of Anesthesiology and Pain Medicine, Asan Medical Center, University of Ulsan College of Medicine, Seoul 05505, Republic of Korea; Department of Anesthesiology and Pain Medicine, Hangang Sacred Heart Hospital, Hallym University College of Medicine, Seoul 07247, Republic of Korea; Department of Anesthesiology and Pain Medicine, Asan Medical Center, University of Ulsan College of Medicine, Seoul 05505, Republic of Korea; Department of Anesthesiology and Pain Medicine, National Medical Center, Seoul 04564, Republic of Korea; Department of Anesthesiology and Pain Medicine, National Medical Center, Seoul 04564, Republic of Korea; Department of Anesthesiology and Pain Medicine, CHA Gangnam Medical Center, CHA University School of Medicine, Seoul 06135, Republic of Korea; Department of Anesthesiology and Pain Medicine, Asan Medical Center, University of Ulsan College of Medicine, Seoul 05505, Republic of Korea

**Keywords:** Red cell distribution width, Albumin ratio, Mortality, Mortality, Burn, Risk factor, Surgery

## Abstract

**Background:**

Red cell distribution width (RDW) and serum albumin concentration are associated with postoperative outcomes. However, the usefulness of the RDW/albumin ratio in burn surgery remains unclear. Therefore, we evaluated the association between RDW/albumin ratio and 90-day mortality after burn surgery.

**Methods:**

Between 2013 and 2020, a retrospective review of patients in a burn intensive care unit (ICU) was performed. Receiver operating characteristic curve, multivariate Cox logistic regression, multivariate logistic regression and Kaplan–Meier analyses were conducted to evaluate the association between RDW/albumin ratio and 90-day mortality after burn surgery. Additionally, prolonged ICU stay rate (>60 days) and ICU stay were assessed.

**Results:**

Ninety-day mortality was 22.5% (210/934) in burn patients. Risk factors for 90-day mortality were RDW/albumin ratio at postoperative day 1, age, American Society of Anesthesiologists physical status, diabetes mellitus, inhalation injury, total body surface area burned, hypotensive event and red blood cell transfusion volume. The area under the curve of the RDW/albumin ratio at postoperative day 1 to predict 90-day mortality, after adjusting for age and total body surface area burned, was 0.875 (cut-off value, 6.8). The 90-day mortality was significantly higher in patients with RDW/albumin ratio >6.8 than in those with RDW/albumin ratio ≤6.8 (49.2% *vs* 12.3%, *p* < 0.001). Prolonged ICU stay rate and ICU stay were significantly higher and longer in patients with RDW/albumin ratio >6.8 than in those with RDW/albumin ratio ≤6.8 (34.5% *vs* 26.5%; 21 [11–38] *vs* 18 [7–32] days).

**Conclusion:**

RDW/albumin ratio >6.8 on postoperative day 1 was associated with higher 90-day mortality, higher prolonged ICU stay rate and longer ICU stay after burn surgery.

Highlights

- This study is the first to show that red cell distribution width (RDW)/albumin ratio could be a prognostic indicator for mortality after burn surgery.
- The 90-day mortality after burn surgery was 22.5% (210/934).
- The RDW/albumin ratio (optimal cut-off value: 6.8) on postoperative day 1 was significantly associated with postoperative 90-day mortality.
- RDW/albumin ratio >6.8 on postoperative day 1 was associated with higher postoperative 90-day mortality, prolonged intensive care unit stay (>60 days) rate and longer intensive care unit stay after burn surgery.

## Background

Burn injury can induce multisystem stress and uncontrolled systemic inflammation, leading to mortality and morbidity [1]. Though the burn mortality rate has declined during the last 5 decades due to improved resuscitation, antibiotic therapy, surgical procedures and nutritional support [2], the mortality rate of burn patients (range 1.4–18%) remains high when compared with that of other critical care patients [3]. Therefore, the prediction of disease progression and mortality is important for improving postoperative outcomes in burn patients.

The red cell distribution width (RDW) is used to easily diagnose and classify anaemia, and is associated with systemic inflammation and several disorders, such as cardiovascular disease, venous thromboembolism, cancer, diabetes mellitus, community-acquired pneumonia, chronic obstructive pulmonary disease and liver and kidney failures [4]. There is a significant association between RDW and mortality and morbidity in diverse conditions, such as trauma [5] and burn injury [6]. The serum albumin concentration reflects host nutritional and inflammatory status [7] and is associated with the prognosis of burn patients [8]. Therefore, we speculated that the combination marker—RDW/albumin ratio—could be a better prognostic indicator for mortality after burn surgery compared with the individual biomarkers. Additionally, we speculated that markers in the postoperative inflammatory laboratory data could reflect surgical stress more precisely than those in the preoperative inflammatory data.

This study was conducted to investigate the association between the RDW/albumin ratio and 90-day mortality after burn surgery. In addition, prolonged intensive care unit (ICU) stay (>60 days) and duration of ICU stay were evaluated.

## Methods

### Patients

This study was performed after the study protocol was approved by the Institutional Research Ethics Board (IREB) of Hangang Sacred Heart Hospital, Hallym University, Republic of Korea (approval number: HG 2021-013). A waiver of the informed consent requirement was approved by the IREB due to the retrospective nature of the study. Computerized chart reviews were performed for patients aged ≥18 years who were admitted to the burn ICU from January 2013 to December 2020. ICU admission was determined by our burn centre admission criteria [8]. Exclusion criteria included incomplete clinical data or follow-up loss. For patients who underwent several burn operations, data from the first operation were assessed.

### Burn care

All burn patients in the ICU received initial fluid management in accordance with a modified Parkland formula of 4 mL/kg/% total body surface area (TBSA). The fluid volume was modified as required to maintain a urine output *>*0.5 mL/kg/h. Patients with serum albumin concentrations of 2.5–2.6 and ≤2.4 mg/dL received 100 and 200 mL of 20% albumin, respectively, according to the Korea Health Insurance Review and Assessment Service (Internal Reduction) Standards. Wound dressing was performed daily using topical antimicrobials and hydrofoam.

### Anaesthetic technique

Anaesthesia was administered as per our standard clinical practice [9], with propofol for the induction and rocuronium for muscle relaxation. Anaesthesia was maintained with the inhalation agents desflurane or sevoflurane. A balanced crystalloid solution was used at a flow rate of 6–10 mL/kg/h. A synthetic colloid solution was used when blood loss exceeded 500 mL. Red blood cell (RBC) transfusion was undertaken when serum haemoglobin concentrations were <8 g/dL. The mean arterial blood pressure (MABP) was maintained at ≥65 mmHg. Additional intravenous fluids or vasoactive drugs were administered when the MABP was <65 mmHg for at least 5 min.

### Surgical technique

Burn surgeries included eschar excision, escharotomy and closure with an allograft or a split-thickness skin graft (STSG). When the burn eschar circumferentially encircled any body part, emergent escharotomy was conducted to reduce the interstitial pressure. According to the burn depth, fascial or tangential excision was performed based on the operator’s decision. An STSG was applied for permanent and rapid closure of full-thickness burns. However, patients with extensive burn wounds were initially covered with an allograft for ~2 weeks and then subsequently covered with an STSG. When deep tissue infection was suspected, extensive wound debridement followed by aseptic dressing with silver sulfadiazine was performed.

### Data collection

The following demographics and preoperative variables were collected: age, sex, body mass index, American Society of Anesthesiologists (ASA) physical status, medical comorbidity, presence of inhalation injury, TBSA burned, area of deep burns requiring excision and skin grafting, type of burn, duration between burn and operation, and preoperative variables (serum haemoglobin concentration, RDW, serum albumin concentration, RDW/albumin ratio and serum creatinine concentration). Preoperative variables were measured within 1 day before surgery. Inhalation injury was clinically determined based on a history of burn in an enclosed space, physical findings (singed facial hairs, facial burn and carbonaceous sputum), bronchoscopic findings (airway oedema, soot in the airway, mucosal necrosis and bronchial ulceration) and elevated carboxyhaemoglobin concentration [8]. The burn size and depth were assessed by a review of the burns recorded in the electronic medical records by burn surgeons who were trained for >10 years.

Intraoperative and postoperative variables were collected and included: operation time, intraoperative hypotensive event, crystalloid infusion amount, colloid infusion amount, RBC transfusion rate, RBC transfusion volume, estimated blood loss and laboratory data on postoperative day 1 including RDW, serum albumin concentration and the RDW/albumin ratio. Postoperative outcomes included 90-day mortality, prolonged ICU stay (>60 days) rate and ICU stay.

### Statistical analysis

Data were tested for normality using the Shapiro–Wilk test. Continuous data are presented as mean ± standard deviation or median (interquartile range) and categorical data are presented as number (percentage). Continuous data were compared using Student’s *t*-test or Mann–Whitney *U* test, as appropriate. Categorical data were compared using the chi-square or Fisher’s exact test, as appropriate. Receiver operating characteristic (ROC) curve analysis was performed to evaluate the predictive abilities of preoperative and postoperative RDW, albumin and RDW/albumin ratio values for 90-day mortality after burn surgery. Moreover, multivariate ROC curve analysis was performed to improve the predictive ability by analyses adjusted for age and TBSA burned. Multivariate Cox proportional hazard regression analysis was performed to evaluate the risk factors of postoperative 90-day mortality in burn patients. Multivariate logistic regression analysis was performed to evaluate the risk factors for a prolonged ICU stay rate after burn surgery. All covariables with *p* < 0.05 in the univariate Cox proportional hazard regression and logistic analyses were included in the multivariate Cox proportional hazard and logistic regression analyses using a forward conditional method. Multicollinearity was assessed by examining the variance inflation factor. Postoperative 90-day survivals between the two groups, which were dichotomized by an RDW/albumin ratio cut-off value, were compared using Kaplan–Meier analysis with a log-rank test. Furthermore, intergroup (groups dichotomized by an RDW/albumin ratio cut-off value) differences in the prolonged ICU stay (>60 days) rate and ICU stay were recorded. *P* < 0.05 was considered statistically significant. All statistical analyses were conducted using Medcalc (version 11.3.3.0; Mariakerke, Belgium), Sigmaplot (version 12.0; Systat Software Inc., Chicago, IL, USA) and SPSS for Windows (version 27.0; IBM-SPSS Inc., Armonk, NY, USA).

## Results

**Figure 1.**
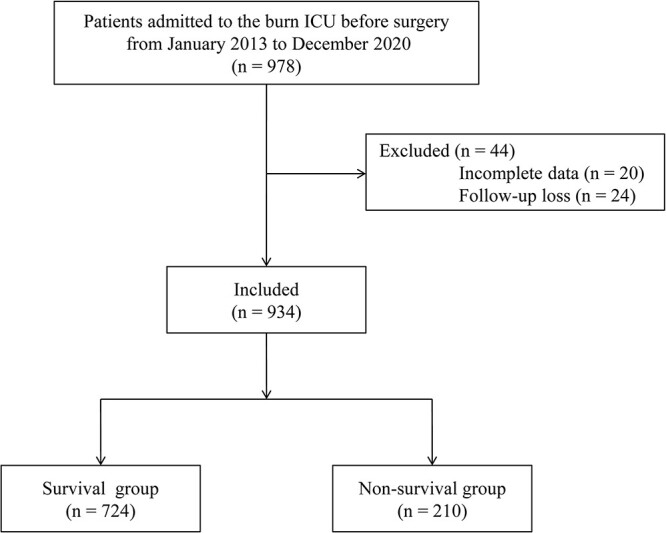
Flowchart of study group is shown. *ICU* intensive care unit

We excluded 44 of the 978 patients who were admitted to the burn ICU between January 2013 and December 2020: 20 patients were excluded due to incomplete data and 24 were excluded due to loss to follow-up. Thus, 934 patients were included in the final analysis. The postoperative 90-day mortality was 22.5% (Figure 1). The causes of death were multiorgan failure (54.3%), septic shock (31.9%), cardiogenic disease (7.6%), acute respiratory infection (5.2%) and cerebrovascular disease (1.0%).

**Figure 2.**
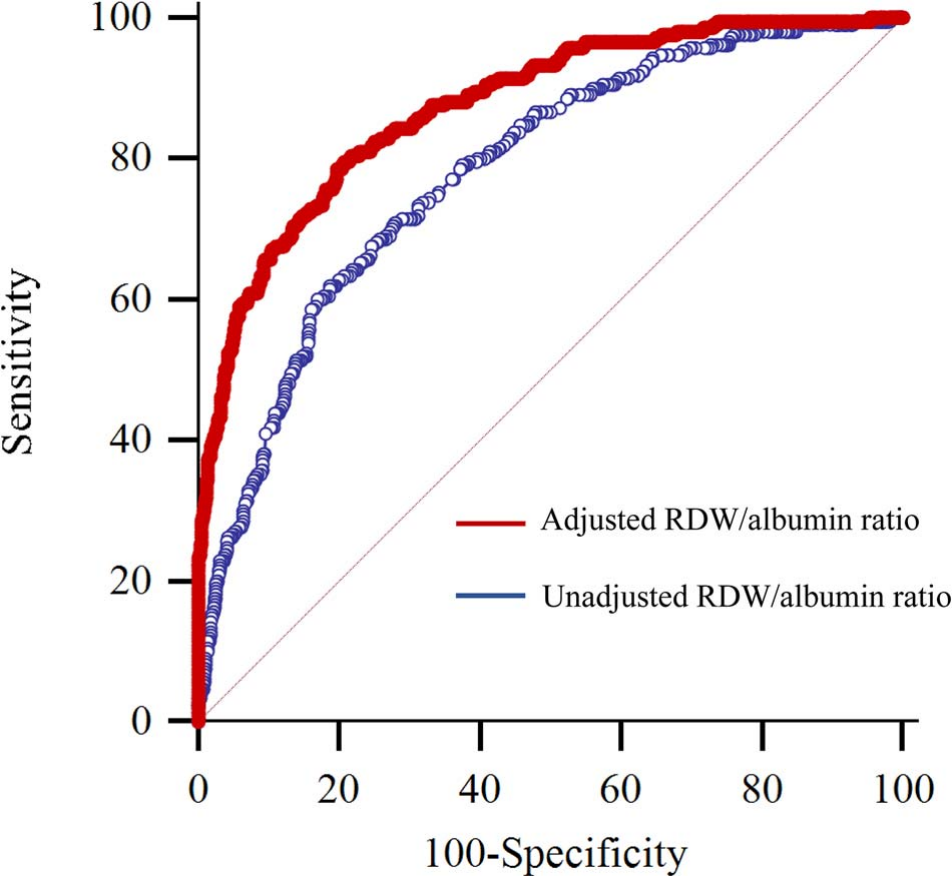
Receiver operating characteristic curve analyses of the unadjusted and adjusted RDW/albumin ratio values on postoperative day 1 to predict postoperative 90-day mortality in burn patients. The RDW/albumin ratio on postoperative day 1 was adjusted for age and the total body surface area burned. The unadjusted and adjusted RDW/albumin ratio AUCs differed significantly. *RDW* red cell distribution width, *AUC* area under the curve

Patient characteristics and laboratory variables were stratified according to 90-day mortality (Tables 1 and 2). The survival and non-survival groups had significantly different RDW/albumin ratios on postoperative day 1. Table 3 shows the ROC curve analyses of preoperative and postoperative RDW, albumin and RDW/albumin ratio values without adjusting and after adjusting for the two important predictors (age and TBSA burned) for postoperative 90-day mortality. In the multivariate ROC curve analysis after adjusting for age and TBSA burned, the area under the curve (AUC) of RDW/albumin on postoperative day 1 was the highest among the biomarkers (AUC: 0.875, 95% confidence interval [CI]: 0.852–0.895; Table 3). The RDW/albumin ratio cut-off value on postoperative day 1 to predict postoperative 90-day mortality was 6.8 (Figure 2).

**Table 1:** Demographics and preoperative variables in 934 patients

**Variables**	**Survival group** **(*n* = 724)**	**Non-survival group** **(*n* = 210)**	***P* value**
Age, years	51 (41–62)	58 (48–70)	<0.001
Sex, male	580 (80.1)	164 (78.1)	0.559
Body mass index, kg/m^2^	23.4 (21.4–25.6)	23.4 (21.4–25.3)	0.625
ASA physical status			<0.001
≤2	353 (48.8)	37 (17.6)	
≥3	371 (51.2)	173 (82.4)	
Diabetes mellitus	61 (8.4)	39 (18.6)	<0.001
Hypertension	145 (20.0)	60 (28.6)	0.010
Ischaemic heart disease	27 (3.7)	9 (4.3)	0.839
Congestive heart failure	8 (1.1)	2 (1.0)	>0.999
Cerebrovascular accidents	30 (4.1)	9 (4.3)	>0.999
Inhalation injury	220 (30.4)	121 (57.6)	<0.001
TBSA burned, %	30.0 (19.0–41.0)	60.0 (40.0–80.0)	<0.001
Area of deep burns requiring excision and skin grafting	29.0 (17.0–41.0)	60.0 (40.0–80.0)	<0.001
Burn type			<0.001
Flame burn	470 (64.9)	183 (87.1)	
Electrical burn	123 (17.0)	5 (2.4)	
Other burns^a^	131 (18.1)	22 (10.5)	
Duration between burn and operation, days	4 (2–7)	3 (2–5)	<0.001
Preoperative laboratory data^b^			
Haemoglobin, g/dL	12.4 (10.4–14.9)	12.8 (10.2–16.3)	0.126
RDW, %	13.0 (12.4–14.0)	13.6 (13.0–14.6)	<0.001
Albumin, g/dL	2.7 (2.3–3.0)	2.3 (1.9–2.7)	<0.001
RDW/albumin ratio, %/g/dL	5.0 (4.3–5.8)	6.2 (5.2–7.3)	<0.001
Creatinine, mg/dL	0.7 (0.5–0.9)	0.9 (0.7–1.2)	<0.001

Data are shown as the median (interquartile range) or the number of patients (%), as appropriate. *ASA* American Society of Anesthesiologists, *TBSA* total body surface area, *RDW* red cell distribution width. ^a^Other burns included scalding burn, contact burn, chemical burn and spark burn. ^b^Preoperative laboratory data were measured within 1 day before surgery

**Table 2:** Intraoperative and postoperative variables in 934 patients

**Variables**	**Survival group** **(*n* = 724)**	**Non-survival group** **(*n* = 210)**	***P* value**
Operation time, min	80 (60–110)	90 (70–126)	<0.001
Hypotensive event	225 (31.1)	133 (63.3)	<0.001
Crystalloid amount, mL/kg	15.4 (9.2–23.9)	20.6 (14.3–29.5)	<0.001
Colloid amount, mL/kg	8.3 (5.0–13.4)	9.7 (5.1–15.9)	0.009
RBC transfusion rate	585 (80.8)	199 (94.8)	<0.001
RBC transfusion volume, unit	3(2–5)	5 (3–7)	<0.001
Estimated blood loss, mL	800 (500–1200)	1200 (800–2000)	<0.001
Laboratory data on postoperative day 1			
RDW, %	13.1 (12.5–13.8)	13.8 (13.1–14.6)	<0.001
Albumin, g/dL	2.4 (2.1–2.8)	1.9 (1.5–2.3)	<0.001
RDW/albumin ratio, %/g/dL	5.5 (4.7–6.5)	7.3 (6.0–9.4)	<0.001

Data are shown as the median (interquartile range) or the number of patients (%), as appropriate. A hypotensive event was defined as a mean arterial blood pressure <65 mmHg for ≥5 min. *RBC* red blood cell, *RDW* red cell distribution width

**Table 3:** ROC curve analyses of preoperative and postoperative RDW, albumin and RDW/albumin ratio values without and with adjustment for age and total body surface area burned for postoperative 90-day mortality

**Variables**	**Unadjusted** **AUC (95% CI)**	**Adjusted^a^** **AUC (95% CI)**	**Difference** **between areas**	***P* value**
Variables within 1 day before surgery				
RDW	0.594 (0.561–0.625)	0.867 (0.844–0.888)	0.273 (0.224–0.323)	<0.001
Albumin	0.679 (0.648–0.709)	0.859 (0.835–0.881)	0.180 (0.139–0.220)	<0.001
RDW/albumin ratio	0.716 (0.685–0.744)	0.863 (0.839 –0.884)	0.147 (0.106–0.189)	<0.001
Variables on postoperative day 1				
RDW	0.680 (0.649–0.710)	0.871 (0.848–0.892)	0.191 (0.143–0.240)	<0.001
Albumin	0.749 (0.719–0.776)	0.868 (0.845–0.889)	0.120 (0.086–0.152)	<0.001
RDW/albumin ratio	0.782 (0.754–0.808)	0.875 (0.852–0.895)	0.093 (0.057–0.128)	<0.001

*ROC* receiver operating characteristic, *RDW* red cell distribution width, *AUC* area under curve, *CI* confidence interval

^a^Variables were adjusted for age and total body surface area burned

Multivariate Cox proportional hazard regression analysis showed that the postoperative 90-day mortality was significantly associated with the RDW/albumin ratio on postoperative day 1, age, ASA physical status, diabetes mellitus, inhalation injury, TBSA burned, hypotensive event and RBC transfusion volume (Table 4). All variance inflation factors were <10.

**Figure 3.**
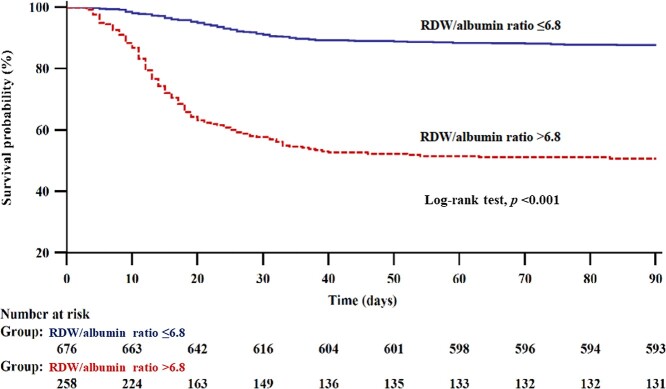
Kaplan–Meier survival analysis of the postoperative 90-day survival rate. Survival was significantly lower in patients with RDW/albumin ratio >6.8 than in those with RDW/albumin ratio ≤6.8. *RDW* red cell distribution width

After categorizing burn patients according to the cut-off value, Kaplan–Meier analysis demonstrated significantly different 90-day survival rate between patients with RDW/albumin ratio >6.8 and ≤6.8 (log-rank test, *p* < 0.001; Figure 3).

In addition, 90-day mortality and the prolonged ICU stay (>60 days) rate after burn surgery were significantly higher in patients with an RDW/albumin ratio >6.8 than in those with RDW/albumin ratio ≤6.8 (127/258 [49.2%] *vs* 83/676 [12.3%], *p* < 0.001; (89/258 [34.5%] *vs* 179/676 [26.5%], *p* = 0.019; Table 5). ICU stay was significantly longer in patients with an RDW/albumin ratio >6.8 than in those with RDW/albumin ratio ≤6.8 (21 (11–38) days *vs* 18 (7–32) days, *p* < 0.001; Table 5). Multivariate logistic regression analysis showed that prolonged ICU stay (>60 days) after burn surgery was significantly associated with RDW/albumin ratio at postoperative day 1, age, diabetes mellitus and inhalation injury (Table 6). There were no new diagnoses of disease or trauma within 90 days after burn surgery.

**Table 4:** Univariate and multivariate Cox proportional hazard regression analyses of risk factors associated with 90-day mortality after burn surgery in 934 patients

**Variables**	**Univariate analysis**		**Multivariate analysis**	
	**HR (95% CI)**	***P* value**	**HR (95% CI)**	***P* value**
Age	1.018 (1.009–1.026)	<0.001	1.050 (1.038–1.062)	<0.001
Sex, male	0.906 (0.654–1.257)	0.556		
Body mass index	1.015 (0.976–1.055)	0.462		
ASA physical status				
≤2	1.000		1.000	
≥3	3.886 (2.724–5.543)	<0.001	1.916 (1.331–2.758)	<0.001
Diabetes mellitus	2.090 (1.476–2.959)	<0.001	2.262 (1.562–3.277)	<0.001
Hypertension	1.489 (1.104–2.009)	0.009		
Ischaemic heart disease	1.109 (0.569–2.162)	0.761		
Inhalation injury	2.672 (2.032–3.514)	<0.001	1.639 (1.223–2.197)	0.001
TBSA burned	1.050 (1.044–1.056)	<0.001	1.051 (1.043–1.059)	<0.001
Burn type				
Flame burn	1.000			
Electrical burn	0.121 (0.050–0.295)	<0.001		
Other burns^a^	0.472 (0.303–0.734)	0.001		
Duration between burn and operation	0.976 (0.954–0.999)	0.040		
Haemoglobin	1.050 (1.007–1.095)	0.023		
Creatinine	1.009 (0.983–1.036)	0.503		
Operation time	1.005 (1.002–1.008)	<0.001		
Hypotensive event	3.204 (2.419–4.243)	<0.001	1.535 (1.149–2.052)	0.004
RBC transfusion volume	1.202 (1.161–1.245)	<0.001	1.055 (1.005–1.107)	0.030
RDW/albumin ratio on postoperative day 1	1.226 (1.194–1.259)	<0.001	1.140 (1.095–1.186)	<0.001

*HR* hazard ratio, *CI* confidence interval, *ASA* American Society of Anesthesiologists, *TBSA* total body surface area, *RBC* red blood cell, *RDW* red cell distribution width. All covariates (i.e. age, ASA physical status, diabetes mellitus, hypertension, inhalation injury, TBSA burned, burn type, duration between burns and operation, haemoglobin, operation time, hypotensive event, RBC transfusion volume and RDW/albumin ratio at postoperative day 1) with *p* < 0.05 in univariate Cox proportional hazard regression analysis were included in the multivariate Cox proportional hazard regression analysis using the forward conditional method. ^a^Other burns included scalding burn, contact burn, chemical burn and spark burn

## Discussion

We found that the RDW/albumin ratio on postoperative day 1 was an important predictor for postoperative 90-day mortality in burn patients. Burn trauma, burn-combined tissue infections and various surgical procedures can incite release of inflammatory mediators that cause intravascular hypovolemia, activate a systemic response that changes pulmonary, cardiovascular, renal, endocrine, hepatic and gastrointestinal functions, and eventually cause death [10]. Burn inflammatory markers, which can readily be evaluated by simple laboratory tests, could be a more specific, convenient and reliable index for mortality when compared with a statistically derived burn severity score calculated by physicians. Therefore, it is useful to evaluate more specific, convenient and economical biochemical markers without additional blood sampling in burn management. RDW is a routine component of a complete blood count (CBC) test that demonstrates heterogeneity in erythrocyte size. This is used as a laboratory parameter for anaemia [4]. Some reports have shown that RDW is associated with inflammation indices, such as erythrocyte sedimentation rate and C-reactive protein [11], suggesting that RDW can be used as an inflammatory index. RDW is greatly influenced by the life span of RBCs; its half-life span is ~4 months. Therefore, RDW can reflect the long-term inflammatory reaction. Furthermore, elevated RDW is associated with poor outcomes in populations with cardiovascular disease [12].

Serum albumin, which is routinely assessed in hospitalized patients, can play an important role in the acute inflammatory response [7]. It has been suggested as a good predictive factor of outcomes in burn patients [8]. There are some controversial results from hypoalbuminaemia and albumin supplementation in patients with major burn [13]; however, postoperative hypoalbuminaemia is associated with mortality [8]. A reduction in serum albumin concentration appears quickly after surgery; it will decline to its minimum level on postoperative day 1 and then reach a stable level over the following 2 days [14]. Early post-surgical albumin reductions develop due to blood loss, altered metabolism and surgical stress [15].

**Table 5:** Postoperative outcomes after burn surgery in 934 patients dichotomized according to the optimal RDW/albumin ratio cut-off value

**Variables**	**RDW/albumin ratio ≤ 6.8** **on postoperative day 1** **(*n* = 676)**	**RDW/albumin ratio > 6.8** **on postoperative day 1** **(*n* = 258)**	***P* value**
90-day mortality	83 (12.3)	127 (49.2)	<0.001
Prolonged ICU stay (>60 days)	179 (26.5)	89 (34.5)	0.019
ICU stay (days)	18 (7–32)	21 (11–38)	<0.001

*RDW* red cell distribution width, *ICU* intensive care unitData are presented as the median (interquartile range) or the number of patients (%), as appropriate

**Table 6:** Univariate and multivariate logistic regression analyses of risk factors associated with prolonged intensive care unit stay (>60 days) after burn surgery in 934 patients

**Variables**	**Univariate analysis**		**Multivariate analysis**	
	**OR (95% CI)**	***P* value**	**OR (95% CI)**	***P* value**
Age	1.023 (1.008–1.039)	0.003	1.023 (1.005–1.040)	0.010
Sex, male	0.659 (0.374–1.162)	0.149		
Body mass index	0.940 (0.870–1.015)	0.116		
ASA physical status				
≤2	1.000			
≥3	1.111 (0.666–1.854)	0.687		
Diabetes mellitus	2.709 (1.461–5.025)	0.002	2.342 (1.219–4.499)	0.011
Hypertension	1.735 (1.008–2.986)	0.047		
Ischaemic heart disease	2.793 (1.119–6.973)	0.028		
Inhalation injury	2.074 (1.254–3.429)	0.004	2.025 (1.185–3.461)	0.010
TBSA burned	1.012 (1.001–1.022)	0.033		
Burn type				
Flame burn	1.000			
Electrical burn	0.087 (0.012–0.637)	0.016		
Other burns^a^	0.859 (0.438–1.685)	0.659		
Duration between burn and operation	1.022 (0.994–1.050)	0.121		
Haemoglobin	0.981 (0.905–1.064)	0.647		
Creatinine	1.000 (0.924–1.082)	0.999		
Operation time	1.003 (0.997–1.009)	0.293		
Hypotensive event	1.049 (0.629–1.752)	0.854		
RBC transfusion volume	1.098 (1.017–1.185)	0.017		
RDW/albumin ratio on postoperative day 1	1.128 (1.049–1.212)	0.001	1.111 (1.027–1.201)	0.008

*OR* odds ratio, *CI* confidence interval, *ASA* American Society of Anesthesiologists, *TBSA* total body surface area, *RBC* red blood cell, *RDW* red cell distribution width. All covariates (i.e. age, diabetes mellitus, hypertension, ischaemic heart disease, inhalation injury, TBSA burned, burn type, RBC transfusion volume and RDW/albumin ratio on postoperative day 1) with *p* < 0.05 in univariate regression analysis were included in the multivariate regression analysis using the forward conditional method. ^a^Other burns included scalding burn, contact burn, chemical burn and spark burn

Taken together, the combination of RDW and albumin can be more closely associated with outcomes in specific clinical conditions. In our study, the AUC of RDW/albumin on postoperative day 1, after adjusting for age and TBSA burned, for 90-day mortality was highest among the preoperative and postoperative RDW, albumin and RDW/albumin ratio values. The combined biomarker of RDW and albumin seems to reflect chronic inflammation, organ dysfunction and acute perioperative stress more precisely than each individual biomarker. Yoo *et al.* have reported that an increased RDW/albumin ratio is associated with mortality in acute respiratory distress syndrome [16]. Furthermore, the RDW/albumin ratio at postoperative day 1 reflects the inflammation and stress during surgery more specifically than the preoperative RDW/albumin ratio. Therefore, we believe that a combined RDW and albumin biomarker on postoperative day 1 has better predictive ability for postoperative 90-day mortality in burn patients.

In the present study, the RDW/albumin ratio cut-off value at postoperative day 1 to predict 90-day mortality was 6.8, which was higher than those in other reports (4.59 in acute respiratory distress syndrome cohorts [16] and 4.94 in post-stroke patients [17]). This difference could be because the decrease in albumin concentration was more pronounced than the increase in RDW in burn patients. In our study, the RDW of the non-survival group was slightly higher than that in the survival group (13.6% *vs* 13.0%). Conversely, the serum albumin concentration of the non-survival group was much lower than that in the survival group (2.3 mg/dL *vs* 2.7 mg/dL). Therefore, we considered that the best cut-off value of RDW/albumin ratio in burn patients may be relatively higher than in other cohorts.

Our study is the first to demonstrate that burn patients with an RDW/albumin ratio >6.8 are at an increased risk of postoperative 90-day mortality. Unlike other risk factors, such as age, ASA physical status, diabetes mellitus, inhalation injury and TBSA burned, which were derived by our Cox regression analysis, the RDW/albumin ratio is a modifiable factor in the perioperative period. Therefore, regular perioperative laboratory examination is recommended to accurately check the serum albumin concentration in burn patients. More active albumin administration during the perioperative period is recommended in burn patients with hypoalbuminaemia. In addition, RDW typically increases in conditions of ineffective RBC production (iron deficiency, vitamin B_12_ or folate deficiency and haemoglobinopathies), increased RBC destruction and blood transfusion [18]. To prevent this elevation, meticulous monitoring and management should be performed to prevent tissue hypoxia/hypoperfusion and reduce inappropriate RBC transfusion in the perioperative period. In line with previous studies, an increase in RDW was detected immediately, reached its highest level at 24 h and remained significantly higher for at least 48 h after RBC transfusion [19–21]. Therefore, the importance of identifying and managing burn patients with an increased RDW/albumin ratio should be emphasized to reduce mortality.

We demonstrated that high RDW/albumin ratios on postoperative day 1 were significantly associated with prolonged ICU stays (>60 days). In line with our study, a high RDW is associated with longer ICU stays in elderly patients with hip fracture [22] and patients with heart failure [23]. Therefore, careful management is required in burn patients with risk factors, including a high RDW/albumin ratio on postoperative day 1.

In our study, the well-known burn prognostic factors (age, inhalation burn injury and TBSA burned) were associated with postoperative 90-day mortality [24]. Furthermore, diabetes mellitus was associated with postoperative 90-day mortality. Burn injury can cause severe insulin resistance with hyperglycaemia, a key feature of metabolic alterations, which is associated higher mortality and morbidity [25]. Additionally, pre-existing diabetes mellitus increases the risk of infections, delays early recovery from burn injury by autonomic neuropathy and increases mortality and morbidity in burn patients [26]**.**

We found ASA physical status ≥3 was associated with postoperative mortality, consistent with previous reports [8,24]. Therefore, careful perioperative management is required to minimize the risk of poor outcomes in burn patients with an ASA physical status ≥3. Furthermore, intraoperative RBC transfusion and hypotension events were associated with mortality in burn patients after surgery in our study. RBC transfusion is a predictor for infection and mortality in burn patients. [27]. In addition, intraoperative hypotensive events were common and significantly associated with cerebrovascular or major adverse cardiac events for all age groups. Cerebrovascular or major adverse cardiac events in the first 30 postoperative days were elevated by 12% for ≤75 mmHg, 17.0% for ≤65 mmHg and 26.0% for ≤55 mmHg [28]. Therefore, reducing the chance of blood transfusion and intraoperative hypotension would improve morbidity and mortality in burn patients.

This study has several limitations. First, it would have been better if the observation points of the factors associated with mortality were unique in the multivariate Cox proportional hazard regression analysis. However, all variables were not simultaneously measured. Nonetheless, we analysed the risk factors at specific time points to increase predictivity. This might have minimally affected our results. Second, this was a single-centre, retrospective cohort study, causing an inevitable selection bias. However, the sample size was relatively large and it was performed in the Hangang Sacred Heart Hospital, the only university-run burn centre in Korea. Most major burn patients in Korea were managed in our burn centre. Therefore, the bias may be minimal in our study.

## Conclusion

The RDW/albumin ratio on postoperative day 1 was significantly associated with postoperative 90-day mortality in burn patients. The 90-day mortality, prolonged ICU stay (>60 days) rates and days of ICU stay were higher in patients with RDW/albumin ratio >6.8 than in those with RDW/albumin ratio ≤6.8. Taken together, our data show that postoperative evaluation of the RDW/albumin ratio is an easy, economical and useful predictor for evaluating mortality after burn surgery.

## Authors’ contributions

YJS, JY, JYP, JHP, HYK, YGK and YKK designed this research work. YJS, JHP, HYK and YGK collected the data. YJS, JY, JYP, NL, JL and YKK analysed the data, performed the statistical analysis and revised the manuscript. YJS, JY and YKK wrote the manuscript. All authors have discussed the results and reviewed the manuscript.

## Ethics approval and consent to participate

This study was performed after the study protocol was approved by the Institutional Research Ethics Board of Hangang Sacred Heart Hospital, Hallym University, Republic of Korea (approval number: HG 2021-013). A waiver of the informed consent requirement was approved by the Institutional Research Ethics Board due to the retrospective nature of the study.

## Conflict of interest

None declared.

## Data availability

Data is available from the authors upon reasonable request.

## Abbreviations

ASA: American Society of Anesthesiologists; AUC: Area under the curve; CBC: Complete blood count; ICU: Intensive care unit; IREB: Institutional Research Ethics Board; MABP: Mean arterial blood pressure; RBC: Red blood cell; RDW: Red cell distribution width; ROC: Receiver operating characteristic; STSG: Split-thickness skin graft; TBSA: Total body surface area.

## References

[1] Zhang P, Zou B, Liou YC, Huang C. The pathogenesis and diagnosis of sepsis post burn injury. Burns Trauma. 2021;9:tkaa047. 10.1093/burnst/tkaa047. PMC7901709 33654698

[2] Lee KC, Joory K, Moiemen NS. History of burns: the past, present and the future. Burns Trauma. 2014;2(4):169–80. 10.4103/2321-3868.143620. 27574647 PMC4978094

[3] Brusselaers N, Monstrey S, Vogelaers D, Hoste E, Blot S. Severe burn injury in Europe: a systematic review of the incidence, etiology, morbidity, and mortality. Crit Care. 2010;14(5):R188. 10.1186/cc9300. 20958968 PMC3219295

[4] Salvagno GL, Sanchis-Gomar F, Picanza A, Lippi G. Red blood cell distribution width: a simple parameter with multiple clinical applications. Crit Rev Clin Lab Sci. 2015;52(2):86–105. 10.3109/10408363.2014.992064. 25535770

[5] Kong T, Park JE, Park YS, Lee HS, You JS, Chung HS, et al. Usefulness of serial measurement of the red blood cell distribution width to predict 28-day mortality in patients with trauma. Am J Emerg Med. 2017;35(12):1819–27. 10.1016/j.ajem.2017.06.008. 28709714

[6] Guo J, Qin Q, Hu H, Zhou D, Sun Y, Deng A. Red cell distribution width (RDW) as a prognostic tool in burn patients. Clin Lab. 2016;62(10):1973–8. 10.7754/Clin.Lab.2016.160222. 28164525

[7] Don BR, Kaysen G. Serum albumin: relationship to inflammation and nutrition. Semin Dial. 2004;17(6):432–7. 10.1111/j.0894-0959.2004.17603.x. 15660573

[8] Seo YJ, Kong YG, Yu J, Park JH, Kim SJ, Kim HY, et al. The prognostic nutritional index on postoperative day one is associated with one-year mortality after burn surgery in elderly patients. Burns Trauma. 2021;9:tkaa043. 10.1093/burnst/tkaa043. PMC7935376 33709002

[9] Kim HY, Kong YG, Park JH, Kim YK. Acute kidney injury after burn surgery: preoperative neutrophil/lymphocyte ratio as a predictive factor. Acta Anaesthesiol Scand. 2019;63(2):240–7. 10.1111/aas.13255. 30203468

[10] Rae L, Fidler P, Gibran N. The physiologic basis of burn shock and the need for aggressive fluid resuscitation. Crit Care Clin. 2016;32(4):491–505. 10.1016/j.ccc.2016.06.001. 27600122

[11] Lippi G, Targher G, Montagnana M, Salvagno GL, Zoppini G, Guidi GC. Relation between red blood cell distribution width and inflammatory biomarkers in a large cohort of unselected outpatients. Arch Pathol Lab Med. 2009;133(4):628–32. 10.5858/133.4.628. 19391664

[12] Hou H, Sun T, Li C, Li Y, Guo Z, Wang W, et al. An overall and dose-response meta-analysis of red blood cell distribution width and CVD outcomes. Sci Rep. 2017;7:43420. 10.1038/srep43420. 28233844 PMC5324076

[13] Eljaiek R, Heylbroeck C, Dubois MJ. Albumin administration for fluid resuscitation in burn patients: a systematic review and meta-analysis. Burns. 2017;43(1):17–24. 10.1016/j.burns.2016.08.001. 27613476

[14] Labgaa I, Joliat GR, Kefleyesus A, Mantziari S, Schäfer M, Demartines N, et al. Is postoperative decrease of serum albumin an early predictor of complications after major abdominal surgery? A prospective cohort study in a European Centre. BMJ Open. 2017;7(4):e013966. 10.1136/bmjopen-2016-013966. PMC5775466 28391235

[15] Hübner M, Mantziari S, Demartines N, Pralong F, Coti-Bertrand P, Schäfer M. Postoperative albumin drop is a marker for surgical stress and a predictor for clinical outcome: a pilot study. Gastroenterol Res Pract. 2016;2016:8743187. 10.1155/2016/8743187. 26880899 PMC4736779

[16] Yoo JW, Ju S, Lee SJ, Cho YJ, Lee JD, Kim HC. Red cell distribution width/albumin ratio is associated with 60-day mortality in patients with acute respiratory distress syndrome. Infect Dis (Lond). 2020;52(4):266–70. 10.1080/23744235.2020.1717599. 31996066

[17] Zhao N, Hu W, Wu Z, Wu X, Li W, Wang Y, et al. The red blood cell distribution width-albumin ratio: a promising predictor of mortality in stroke patients. Int J Gen Med. 2021;14:3737–47. 10.2147/ijgm.S322441. 34326660 PMC8315287

[18] Oh HJ, Park JT, Kim JK, Yoo DE, Kim SJ, Han SH, et al. Red blood cell distribution width is an independent predictor of mortality in acute kidney injury patients treated with continuous renal replacement therapy. Nephrol Dial Transplant. 2012;27(2):589–94. 10.1093/ndt/gfr307. 21712489

[19] Bazick HS, Chang D, Mahadevappa K, Gibbons FK, Christopher KB. Red cell distribution width and all-cause mortality in critically ill patients. Crit Care Med. 2011;39(8):1913–21. 10.1097/CCM.0b013e31821b85c6. 21532476 PMC4427349

[20] Spadaro S, Taccone FS, Fogagnolo A, Franchi F, Scolletta S, Ragazzi R, et al. The effects of blood transfusion on red blood cell distribution width in critically ill patients: a pilot study. Transfusion. 2018;58(8):1863–9. 10.1111/trf.14759. 29770452

[21] Felker GM, Allen LA, Pocock SJ, Shaw LK, McMurray JJ, Pfeffer MA, et al. Red cell distribution width as a novel prognostic marker in heart failure: data from the CHARM program and the Duke databank. J Am Coll Cardiol. 2007;50(1):40–7. 10.1016/j.jacc.2007.02.067. 17601544

[22] Brown M, Nassoiy S, Plackett T, Luchette F, Posluszny J, Jr. Red blood cell distribution width and outcome in trauma patients. J Osteopath Med. 2021;121(2):221–8. 10.1515/jom-2020-0089. 33567079 PMC8086633

[23] Massin MM . Relation between red cell distribution width and clinical outcome after surgery for congenital heart disease in children. Pediatr Cardiol. 2012;33(7):1021–5. 10.1007/s00246-012-0220-0. 22314369

[24] Yu J, Kim HY, Kong YG, Park JH, Seo YJ, Kim YK. De Ritis ratio as a predictor of 1-year mortality after burn surgery. Burns. 2021;47(8):1865–72. 10.1016/j.burns.2021.02.001. 33832798

[25] Bittner EA, Shank E, Woodson L, Martyn JA. Acute and perioperative care of the burn-injured patient. Anesthesiology. 2015;122(2):448–64. 10.1097/aln.0000000000000559. 25485468 PMC4844008

[26] Yang B, Cai YQ, Wang XD. The impact of diabetes mellitus on mortality and infection outcomes in burn patients: a meta-analysis. Eur Rev Med Pharmacol Sci. 2021;25(6):2481–92. 10.26355/eurrev_202103_25411. 33829434

[27] Palmieri TL, Caruso DM, Foster KN, Cairns BA, Peck MD, Gamelli RL, et al. Effect of blood transfusion on outcome after major burn injury: a multicenter study. Crit Care Med. 2006;34(6):1602–7. 10.1097/01.Ccm.0000217472.97524.0e. 16607231

[28] Gregory A, Stapelfeldt WH, Khanna AK, Smischney NJ, Boero IJ, Chen Q, et al. Intraoperative hypotension is associated with adverse clinical outcomes after noncardiac surgery. Anesth Analg. 2021;132(6):1654–65. 10.1213/ane.0000000000005250. 33177322 PMC8115733

